# Applications of the InChI in cheminformatics with the CDK and Bioclipse

**DOI:** 10.1186/1758-2946-5-14

**Published:** 2013-03-13

**Authors:** Ola Spjuth, Arvid Berg, Samuel Adams, Egon L Willighagen

**Affiliations:** 1 Department of Pharmaceutical Biosciences, Uppsala University, Uppsala, 751 24 Sweden; 2 Unilever Centre for Molecular Sciences Informatics, University Chemical Laboratory Cambridge, CB2 1EW, UK; 3 Department of Bioinformatics - BiGCaT, Maastricht University, Maastricht, NL-6200 MD, The Netherlands

**Keywords:** InChI, InChIKey, Chemical structures, JNI-InChI, The Chemistry Development Kit, OSGi, Bioclipse, Decision support, Linked data, Tautomers, Databases, Semantic web

## Abstract

**Background:**

The InChI algorithms are written in C++ and not available as Java library. Integration into software written in Java therefore requires a bridge between C and Java libraries, provided by the Java Native Interface (JNI) technology.

**Results:**

We here describe how the InChI library is used in the Bioclipse workbench and the Chemistry Development Kit (CDK) cheminformatics library. To make this possible, a JNI bridge to the InChI library was developed, JNI-InChI, allowing Java software to access the InChI algorithms. By using this bridge, the CDK project packages the InChI binaries in a module and offers easy access from Java using the CDK API. The Bioclipse project packages and offers InChI as a dynamic OSGi bundle that can easily be used by any OSGi-compliant software, in addition to the regular Java Archive and Maven bundles. Bioclipse itself uses the InChI as a key component and calculates it on the fly when visualizing and editing chemical structures. We demonstrate the utility of InChI with various applications in CDK and Bioclipse, such as decision support for chemical liability assessment, tautomer generation, and for knowledge aggregation using a linked data approach.

**Conclusions:**

These results show that the InChI library can be used in a variety of Java library dependency solutions, making the functionality easily accessible by Java software, such as in the CDK. The applications show various ways the InChI has been used in Bioclipse, to enrich its functionality.

## Background

It is of great importance that chemical structures can be serialized in standard formats in order to enable exchange and linking of chemical information. The IUPAC Chemical Identifier (InChI) [1] is such a standardized identifier for chemical structures, which lately has seen a great adoption in the cheminformatics community [2]. A recent special issue details this further [3]. Two important use cases are querying for exact matches in databases, and linking chemical structures using semantic web technologies. The official implementation of InChI is in C as a library, in order to provide a single implementation that everyone can use. This however limits its use in other programming languages such as Java. We here describe the packaging of InChI in Java, to enable frameworks and applications written in this language, like the applications mentioned in this paper, BioJava [4], JOELib [5], and JChem [6], to take advantage of the benefits of InChI. We present the integration of InChI in the cheminformatics library the Chemistry Development Kit as well as the graphical workbench Bioclipse. We also provide demonstrations where InChI is used in decision support for chemical liability assessment, for tautomer generation, and for knowledge aggregation using a linked data approach.

## Implementation

### Packaging InChI in Java Archives and Maven bundles

JNI-InChI is the packaging of the InChI libraries in portable Java libraries using the Java Native Interface (JNI), available on Sourceforge under GNU Lesser General Public License 3.0 (LGPL) [7]. The JNI-InChI library provides native binaries of the InChI library for 32- and 64-bit Windows, Linux and Solaris, 64-bit FreeBSD and 64-bit Intel-based Mac OS X, covering the most common platforms on which the CDK and Bioclipse are run. The library is available as a regular Jar Archive (.jar file), as Maven bundle from the JNI-InChI project website at http://jni-inchi.sf.net/.

### Provisioning of InChI as OSGi bundles

While Maven makes library dependency management a lot easier, it is not the only platform to do so. OSGi [8] is another standard for dynamic module system in Java, allowing for easy provisioning and interoperability of modules, mainly containing compiled Java code but also associated data. The Bioclipse project has developed OSGi bundles for InChI by wrapping the JNI-InChI libraries, which required some modifications to e.g. class loaders. The OSGi bundles are available from a p2 repository for easy provisioning and integration. Having OSGi bundles with InChI enables easy access from all plugins supporting this module technology. Cheminformatics tools that makes use of the OSGi module system includes KNIME [9], Cytoscape (as of version 3) [10], Taverna [11,12], and Bioclipse [13]. More information and the bundles can be found at http://www.bioclipse.net/inchi-osgi.

### The JNI-InChI API

The JNI-InChI library is written to directly make calls to the InChI library. That is, it will make library calls directly, rather than using a command line to access the library. To make this possible with JNI, it defines a JniInchiWrapper class which has a Java API of which some methods are written in Java, and some call native methods in the matching JniInchiWrapper.c class that directly calls the C++ InChI library. This wrapper allows the JNI-InChI user to set up a proper data model for the chemical structure for which the InChI should be calculated, and to set the generation options, allowing users to select, for example, which InChI layers should be generated or if just a standard InChI should be calculated.

The code subset of the API of the JniInchiWrapper and JniInchiStructure classes is given in Table 1. Using this API we can, for example, calculate the InChI string for ethane (without non-default options; in Java):

The full API is available as HTML JavaDoc at http://jni-inchi.sourceforge.net/apidocs/. What the API does not do, is support input of chemical structures from chemical file formats, such as the MDL molfile format supported by the InChI library itself. Instead, JNI-InChI encourages cheminformatics libraries to use converters that translate their internal data structure into the JNI-InChI data structure, using the methods of the JniInchiInput class. One library taking this approach is the CDK.

**Table 1:** Various java methods from the JniInChIWrapper class

**JniInChIWrapper**	
loadLibrary()	Loads the InChI library suitable for theplatform.
getInchi(JniInchiInput)	Generates an InChI for the given inputstructure, with the InChI options passedwith the input.
getStdInchi(JniInchiInput)	Generates a Standard InChI for the giveninput structure.
getStructureFromInchi(JniInchiInputInchi)	Generates a structure from an InChI string(without coordinates).
getInchiKey(String)	Converts an InChI into an InChIKey.
checkInchi(String, boolean)	Check the validity of a (non-standard) InChIeither loosely or strict.
checkInchiKey(String, boolean)	Check the validity of a (non-standard)InChIKey either loosely or strict.
**JniInchiInput**	
JniInchiInput(List)	Constructor allowing you to set the InChIgeneration options as a List of Strings.
addAtom(JniInchiAtom)	Adds an atom to the input structure.
addBond(JniInchiBond(	Adds a bond to the input structure.
addStereo0D(JniInchiStereo0D)	Adds a tetrahedral, bond, or allenestereochemistry element to the inputstructure.

### Integration of JNI-InChI into the CDK

The primary purpose of the integration of the JNI-InChI into the CDK is to allow the translation of the CDK data structure into that of JNI-InChI. Using this approach, we can convert the content of any chemical file format the CDK supports into InChIs, overcoming limitations of the InChI library in terms of supported file formats.

While JNI-InChI supports the full range of functionality of the InChI C library, structure-to-InChI, InChI-to-structure, AuxInfo-to-structure, InChIKey generation, and InChI and InChIKey validation, not all of this functionality is available in the CDK library, in version 1.4.13 and later.

The CDK-to-JNI-InChI bridge supports the following layers: the connectivity layer, tetrahedral and double bond stereochemistry layers, the isotope layer, and the charge layer. Additionally, the CDK API for generating InChIs allows the use of various options, so that standard InChIs and non-standard InChIs can be generated. For example, an InChI with the fixed hydrogen layer can be calculated with the Java code:

The CDK uses this functionality further for generate tautomers, as proposed by Thalheim et al. [14], and demonstrated later in this paper. Another feature is that the InChI library can be use to generate canonical atom numbers, which is done with the InChINumbersTools class.

### Integration of InChI in Bioclipse

**Figure 1:**
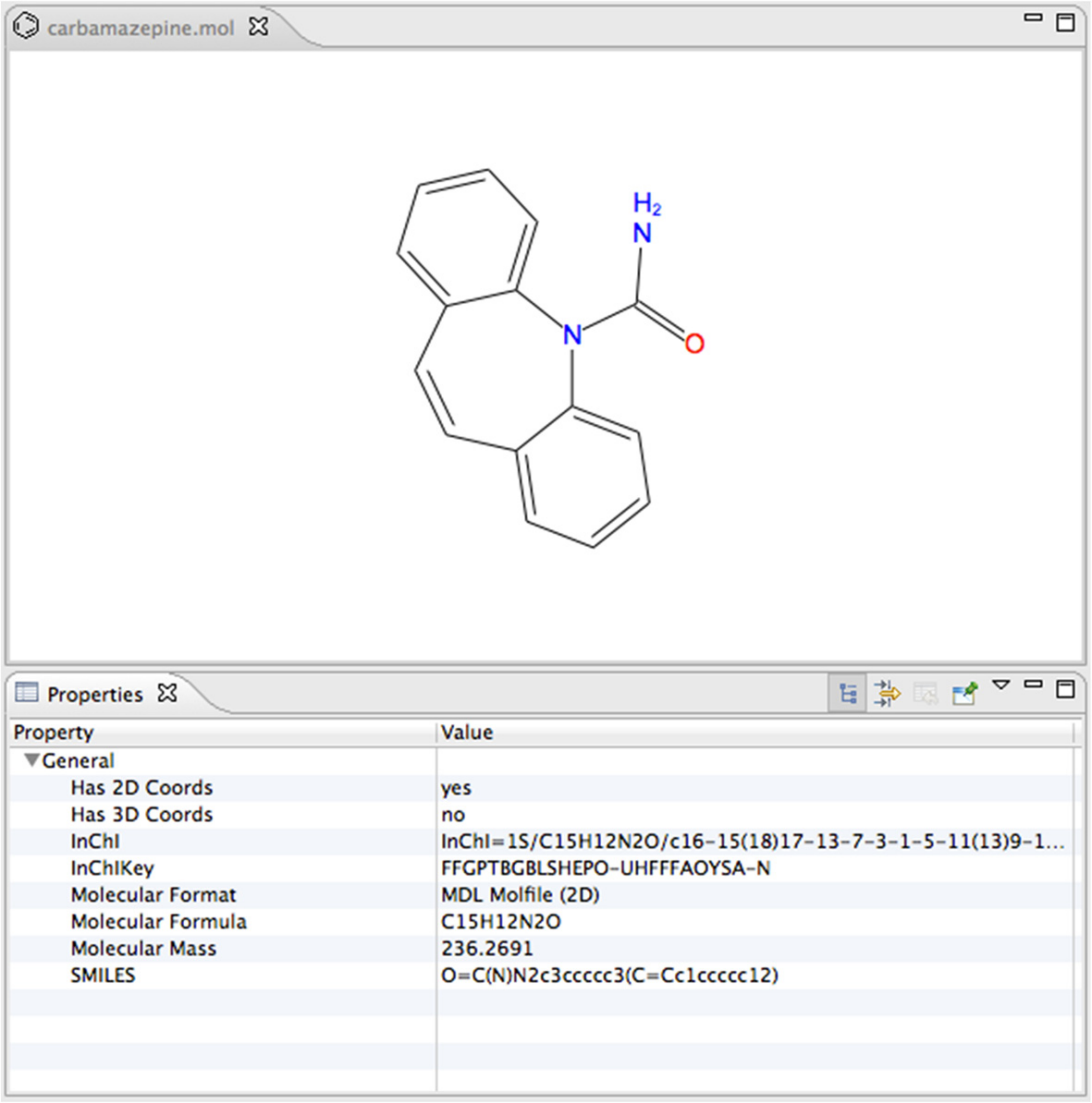
**Part of the Bioclipse workbench showing the chemical structure for the drug carbamazepine.** The InChI and InChIKey are displayed as properties in the bottom canvas. Editing the chemical structure instantly triggers a recalculation of these properties.

Bioclipse is a workbench for the life sciences where cheminformatics is the most developed functionality. Key features of Bioclipse includes import, export and editing of chemical structures in various file formats, as well as visualizations and various property calculations - all features available from both a graphical workbench as well as a built-in scripting language (Bioclipse Scripting Language, or BSL) [15,16] and lately via a link to the statistical programming language R [17]. As a Rich Client built on the Eclipse Rich Client Platform (RCP), Bioclipse inherits an extensible architecture implementing the OSGi standard. By adding the previously described InChI OSGi bundles to Bioclipse, Bioclipse exposes InChI calculation as a key feature in the workbench, and InChI is calculated on all structure modifications and visualized as a general property in the workbench window (see Figure 1). Bioclipse supports both the generation of standard and non-standard InChIs, and a preference allows for selecting between the different versions. An example in BSL is:

## Results and discussion

The applications below have additional information on how to install and perform them available on: http://www.bioclipse.net/inchi.

### Applications of InChI in cheminformatics

#### a) Decision support in computational pharmacology

**Figure 2:**
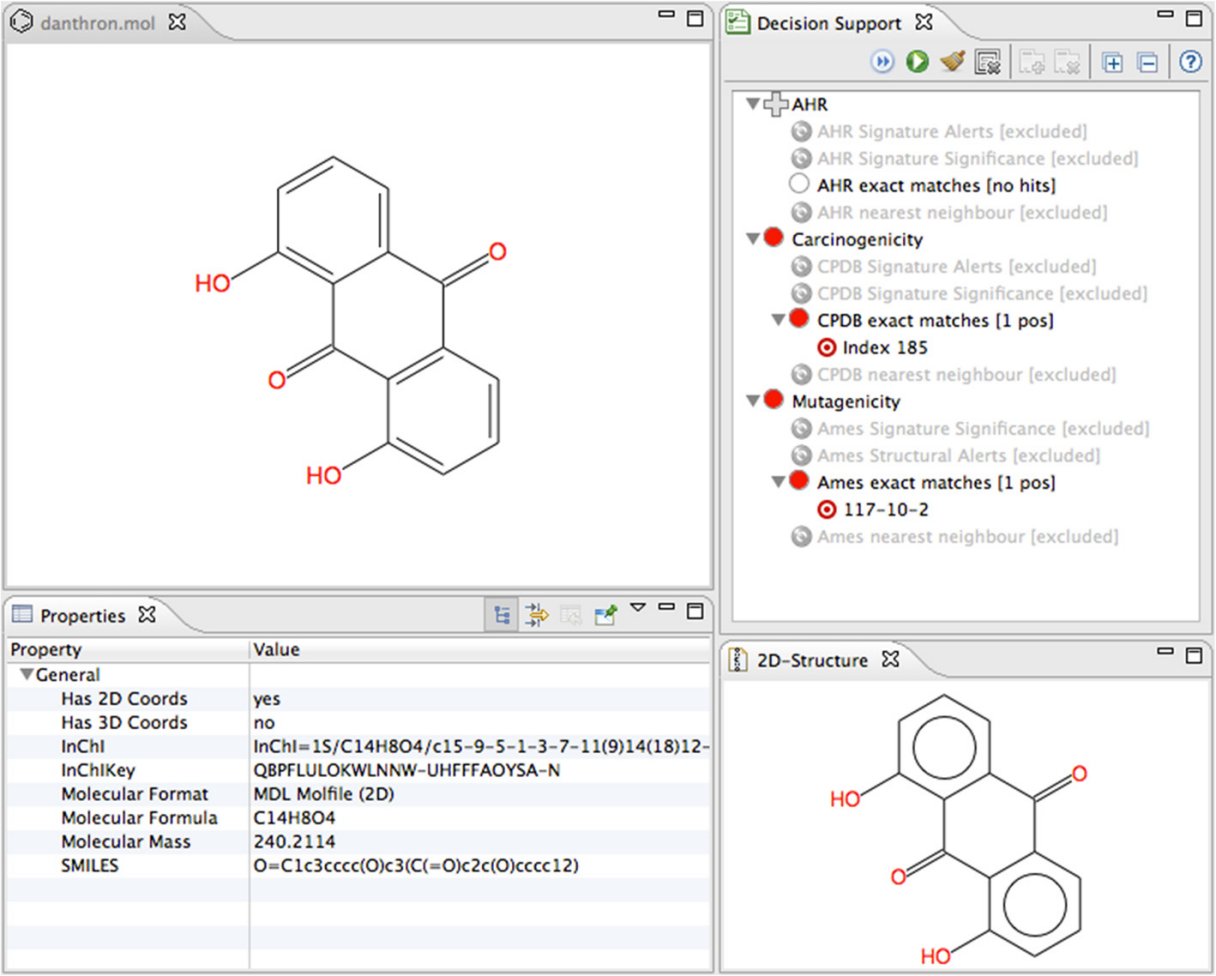
**Part of the Bioclipse workbench showing the Decision Support feature.** It shows three exact matches enabled (right canvas) and the chemical structure of the withdrawn drug danthron. We see that the data sets for CPDB [19] and Ames Mutagenicity [20] both gives an exact match, and that this compound has previously been shown to be positive (mutagen) in an Ames Mutagenicity test as well as positive for an in vivo carcinogenicity test included in the Carcinogenicity Potency Database.

In chemical safety assessment, the first step when faced with a new chemical structure is to see weather it already has been synthesized, and if any in vitro assays or in vivo studies have been performed. Given the large size of knowledge bases in companies and organizations, exact database lookups have become ubiquitous tools and used on a daily basis. Bioclipse Decision Support provides a framework for running exact match queries against a library of chemical structures, which was demonstrated for 3 open safety endpoints [18]. An example query can be seen in Figure 2.

#### b) Linked data spidering in Bioclipse with Isbjørn

Molecular structures on the internet can be searched using InChI and InChIKeys [21] directly. However, they can also be used as seed to spider (the process of following links on the world wide web) the Linked Data section of the World Wide Web [22]. We developed a plugin to Bioclipse that searches the Internet for information about a molecule, initiated with the InChI and a web service we developed earlier, providing Universal Resource Identifiers for molecules, available at http://rdf.openmolecules.net/[23]. This service provides a number of initial links to other Linked Data resources, and links to other resources are followed using owl:sameAs and skos:exactMatch predicates.

**Figure 3:**
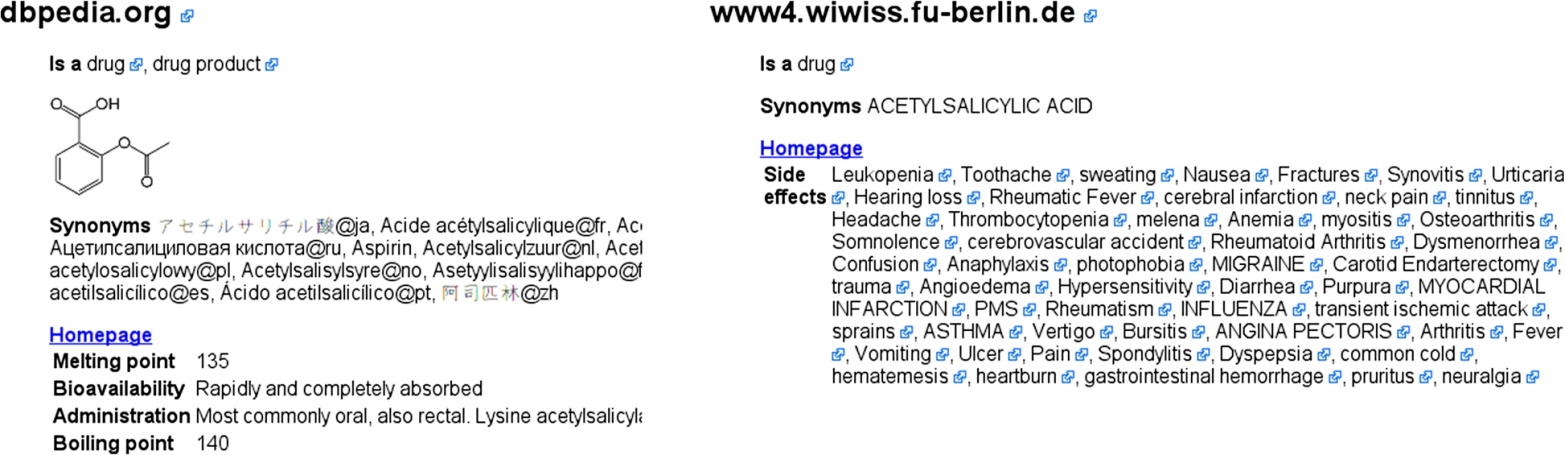
Screenshot of Linked Data spidering results by Isbjørn presented as a HTML page.

While spidering the web of molecular information, common ontologies are recognized and use to extract information about the compound. Recognized ontologies include general ontologies like Dublin Core (http://dublincore.org/), RDF Schema [24], SKOS [25], and FOAF [26], as well as domain specific ontologies, like ChemAxiom [27], CHEMINF [28], and specific predicates used by specific databases, including Bio2RDF [29], DBPedia [30], and ChemSpider [31] (see Figure 3 left).

But by educating Isbjørn about further ontologies we can even, for example, extract drug side effects from the SIDER database [32], as exposed by the Free University Berlin RDF services, as shown in Figure 3 right. The search results of Isbjørn are presented in Bioclipse as a HTML page and opened in a browser window (not shown).

#### c) CDK tautomer calculation in Bioclipse

The InChI library can also be used to generate tautomers [14]. This method has been implemented in the CDK by Rijnbeek [33], and exposed in the Bioclipse Scripting Language. Tautomers can be calculated for any molecule, for example, created from a SMILES string in this example for phenol:

Using this approach we can generate tautomers for any molecules, though it is limited by the heuristic rules implemented by the InChI library. We typically only find a subset of tautomers, rather than a full set. For example, for warfarin it finds only six tautomers out of the 40 reported ones [34].

## Conclusions

The InChI project has chosen the path to rely on a single implementation for standardizing InChI calculations, and it is important that this code is readily available for all cheminformatics software development. This paper describes the packaging of InChI as a Java library using a JNI bridge (JNI-InChI), which is available as a Java Archive (jar file), and as Maven bundles. It further shows the integration into the CDK library and how the JNI-InChI as OSGi bundles renders InChI easily available for software using this dynamic module system, such as the Bioclipse workbench. The various binary packages make the InChI library easily usable in a variety of Java environments.

A feature of the InChI is that it supports various layers of detail in describing the chemical structure, which has confused end users of cheminformatics software. This resulted in a set of chosen layers, resulting in the standard InChI. The CDK supports generation and processing of both the standard and non-standard InChIs. Bioclipse provides a preference page where users can indicate which InChI they like to be calculated by default.

The uses in the CDK and Bioclipse have shown that the InChI is of great utility for uniquely identifying molecular structures in a canonical form, and is therefore well suited for exact matches in database searches, as exemplified in computational pharmacology example. This makes it also highly suitable for mining the internet and the Linked Data network. We demonstrate this with our Isbjørn plugin for Bioclipse, which aggregates knowledge about chemical compounds from an increasing list of disparate sources. The use of the InChI here shows the potential for the common task to collect as much information as possible about a novel chemical structure, uniquely identified by the InChI. But the use of the InChI algorithms is not limited to that purpose, and has further benefits. We demonstrate this with the exposure in the CDK and Bioclipse to generate tautomers.

Our results show that it is possible to overcome the problem that the InChI algorithm is not implemented in Java, but this however comes at a price. Using non-Java code in a Java environment requires a bridge, for which we used JNI, but crossing this bridge is computationally expensive. Furthermore, the integration into the CDK requires bridging two data models: one for the CDK and one for the InChI library. A suite of unit tests is in place to validate that information is correctly translated from the CDK data model into calculated InChIs. However, a full validation using the InChI project test suite has not been completed yet.

## Availability and requirements

● **Project Name**: JNI-InChI

● **Project home page**: http://jni-inchi.sourceforge.net/

● **Operating system(s)**: Windows, GNU/Linux, OS/X

● **Programming language**: C and Java

● **Other requirements (if compiling)**: InChI library

● **License**: GNU LGPL v3 or later

● **Any restrictions to use by non-academics**: None additional

● **Project Name**: The Chemistry Development Kit

● **Project home page**: http://cdk.sourceforge.net/

● **Operating system(s)**: Platform independent

● **Programming language**: Java

● **Other requirements (for the InChI module)**: JNI-InChI

● **License**: GNU LGPL v2.1 or later

● **Any restrictions to use by non-academics**: None additional

● **Project Name**: Bioclipse

● **Project home page**: http://www.bioclipse.net/

● **Operating system(s)**: Windows, GNU/Linux, OS/X

● **Programming language**: Java

● **Other requirements (for InChI functionality)**: JNI-InChI, The Chemistry Development Kit

● **License**: Eclipse Public License

● **Any restrictions to use by non-academics**: None additional

## Competing interests

The authors declare that they have no competing interests.

## Authors’ contributions

OS and EW wrote major parts of the manuscript and organized the paper writing process. SA wrote the JNI-InChI library and the CDK integration. AB created the OSGi bundles. EW wrote the Isbjørn plugin and application. OS, AB, and EW made the InChI functionality available in Bioclipse. The decision support use case was developed by OS. All authors read and approved the final manuscript.
